# Nanostructured Silicon as Potential Anode Material for Li-Ion Batteries

**DOI:** 10.3390/molecules25040891

**Published:** 2020-02-17

**Authors:** Matea Raić, Lara Mikac, Ivan Marić, Goran Štefanić, Marko Škrabić, Marijan Gotić, Mile Ivanda

**Affiliations:** 1 Laboratory for Molecular Physics and Synthesis of New Materials, Ruder Bošković Institute, Bijenička c. 54, 10000 Zagreb, Croatia; matea.raic@irb.hr (M.R.); lmikac@irb.hr (L.M.); Goran.Stefanic@irb.hr (G.Š.); gotic@irb.hr (M.G.); 2 Research Unit New Functional Materials, Center of Excellence for Advanced Materials and Sensing Devices, Bijenička c. 54, 10000 Zagreb, Croatia; 3 Radiation Chemistry and Dosimetry Laboratory, Ruđer Bošković Institute, Bijenička c. 54, 10000 Zagreb, Croatia; imaric@irb.hr; 4 Department of Physics and Biophysics, School of Medicine, University of Zagreb, Šalata 3b, 10000 Zagreb, Croatia; marko.skrabic@mef.hr

**Keywords:** silicon, ball-milling, chemical etching, porosity, anode, battery, electrochemical performance

## Abstract

Commercial micrometer silicon (Si) powder was investigated as a potential anode material for lithium ion (Li-ion) batteries. The characterization of this powder showed the mean particle size of approx.75.2 nm, BET surface area of 10.6 m^2/^g and average pore size of 0.56 nm. Its band gap was estimated to 1.35 eV as determined using UV-Vis diffuse reflectance spectra. In order to increase the surface area and porosity which is important for Li-ion batteries, the starting Si powder was ball-milled and threatened by metal-assisted chemical etching. The mechanochemical treatment resulted in decrease of the particle size from 75 nm to 29 nm, an increase of the BET surface area and average pore size to 16.7 m^2^/g and 1.26 nm, respectively, and broadening of the X-ray powder diffraction (XRD) lines. The XRD patterns of silver metal-assisted chemical etching (MACE) sample showed strong and narrow diffraction lines typical for powder silicon and low-intensity diffraction lines typical for silver. The metal-assisted chemical etching of starting Si material resulted in a decrease of surface area to 7.3 m^2^/g and an increase of the average pore size to 3.44 nm. These three materials were used as the anode material in lithium-ion cells, and their electrochemical properties were investigated by cyclic voltammetry and galvanostatic charge-discharge cycles. The enhanced electrochemical performance of the sample prepared by MACE is attributed to increase in pore size, which are large enough for easy lithiation. These are the positive aspects of the application of MACE in the development of an anode material for Li-ion batteries.

## 1. Introduction

**Figure 1:**
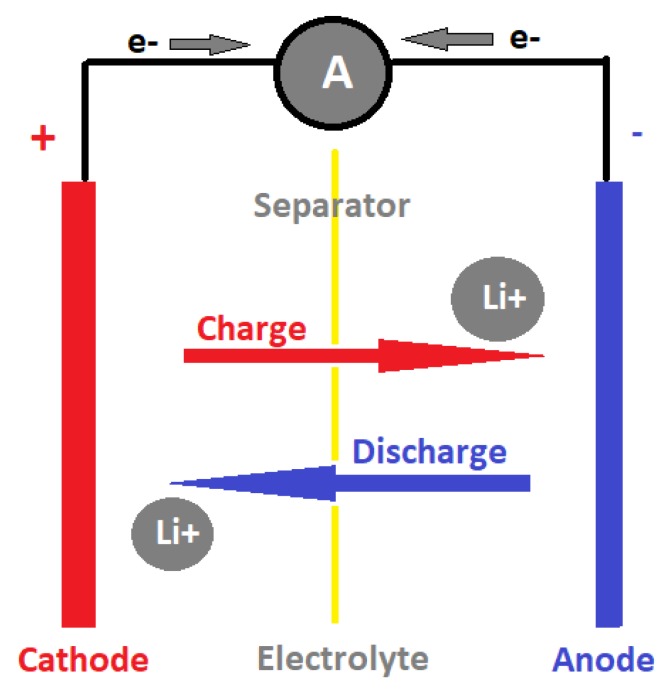
Schematic of a traditional lithium ion (Li-ion) battery cell.

The success of lithium ion (Li-ion) batteries in the early 1960s took years of research and contribution of many scientists and engineers. Since then, have been several electronic revolutions, and Li-ion cells are still the most widely used as a rechargeable battery system for portable electronic devices and electric vehicles. They have many advantages, including high energy density, long storage life, small volume, lightweight, low self-discharge efficiency, and non-memory effect. Rechargeable Li-ion batteries mainly consist of two electrodes—a separator and electrolyte (Figure 1). When the battery is charged, lithium ions released from the positive electrode (cathode) will move toward the negative electrode (anode). When a battery is discharged, lithium ions will move from anode to cathode. Meanwhile, electrons released from lithium atoms in the anode will travel through the external circuit to the cathode, which provides electric power from chemical energy [1].

**Figure 2:**
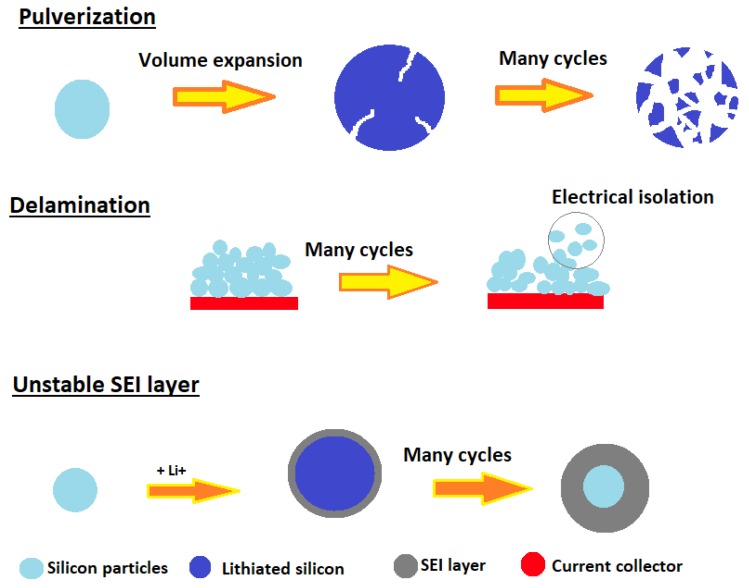
Cell failure mechanisms of silicon [24].

There have been many efforts made to further improve the performance of Li-ion batteries, which achieved certain significant progress. At first, lithium metal was used as the anode, but it contains dendritic Li growth during cycling processes which is known to possess serious safety hazards [2,3]. Later, in 1991, Sony [4] discovered that pyrolytic carbon can effectively insert lithium ions, so they introduced the first commercial Li-ion battery based on C/LiCoO_2_ [5]. Li-ion cells have higher operating voltage and lower self-discharge compared to some conventional secondary cells, such as nickel-cadmium and nickel-metal hydride. Despite the effort made in recent years, a further increase in energy densities (Wh l^−1^) and mass capacities (Wh kg^−1^) are still required to make Li-ion cells usable for low- or zero-hybrid and electric vehicles, energy-efficient cargo ships, locomotives, aerospace, and power-grid applications [6,7,8,9]. By replacing lithium cobalt oxide cathode and carbon anodes with higher performance electrode materials, the devices can be improved [10]. A few alternatives to cathode material have been developed. Many of them are either olivine type phosphates [11,12] layered compounds with hexagonal symmetry based on a α-NaFeO_2_ structure, such as LiNiO_2_, LiNi_x_Co_y_O_2_, etc. Various elements such as Co, Mn, Ni, Cr, Al, or Li can be substituted into the α-NaFeO_2_ structure and influence on stability and electronic conductivity [13,14,15]. The enhancement in the morphology of anode materials leads to better capacitance properties. Nanostructure design is an effective way to improve battery cycling because nanostructures provide short diffusion length for Li^+^ ions and electrons with better resistance to fracture. Si (4200 mAh/g)-, Sn (992 mAh/g)-, and SnO2 (782 mAh/g)-based anodes have high gravimetric and volumetric capacities, so they are the most attractive and widely investigated candidate materials among the different alloys [16,17,18]. The most widely used anode is graphite, whose lithiated compounds have stable phases up to the LiC_6_ stoichiometry, corresponding to a theoretical specific capacity of 372 mAh/g [19]. In contrast, silicon possesses a very high theoretical capacity of 4200 mAh/g and can intercalate 4.4 Li into Si at high temperatures to form Li_15_Si_4_ [20]. Silicon also features a working potential around 0.4 V vs. Li/Li^+^, which is safer than operating potential of graphite (0.05 V vs. Li/Li^+^) [21]. Although silicon possesses all of these advantages, silicon-based anodes suffer from huge volume expansion upon cycling (≈400%) causing electrode fracture and electrical isolation during repeated cycling (Figure 2) [21,22]. Continuous volume changes cause the breaking-reformation of the solid electrolyte interphase (SEI) film, which leads to the consumption of lithium ions and electrolyte. Passivating SEI layer usually contains non-cyclable lithium ions (LiF, Li_2_O), which are trapped by irreversible side reactions. Liquid electrolytes reductively decompose at the working potential of Si (<0.4 V vs. Li/Li^+^), forming the SEI layer on a conductive surface. Solvent exhaustion causes the degradation of conductivity and induces fast capacity loss [21,23].

There are two strategies to avoid this problem. The first is combining Si with different kinds of carbon materials such as amorphous carbon [25,26,27], conductive carbon black [28], carbon nanotubes [29,30], and graphene [31,32,33], and the second is by designing nanoscale silicon with different structures. Currently, extensive research has been carried out to develop nanostructures of silicon i.e., silicon nanoparticles [27,34,35], silicon nanowires/nanotubes [36,37,38], nanosheets [38,39], and 3D porous structures [40,41]. The porous structure can provide a large space to accommodate volume expansion and provide a large surface area for lithium-ion transport from electrolyte to silicon [42]. Porous silicon particles could be prepared by electrochemical etching and subsequent planetary ball milling [28].

In this work, we studied silicon microparticles for their possible use in anode fabrication to produce non-expensive anode material, which can achieve better capacitance properties. Commercial silicon powder was used as a starting material. The same material was ball-milled and chemically etched to obtain nanostructured material. Several works have been published on ball-milled Si nanocomposite anodes [26,43], but in this work, we present pure Si powder as a powerful and low-cost approach for producing Si microparticles. The micrometric size of Si powder allows safe and easy handling. Furthermore, this process could use Si waste (typically Si wafer scraps) as the starting material, reducing the precursor cost to nearly zero. In the present work, structural and microstructural changes in these three materials were examined using several methods–X-ray powder diffraction (XRD) combined with the results of Raman spectroscopy, FT-IR spectroscopy, UV-Vis spectrometry, nitrogen adsorption measurements, and TEM analysis. Electrochemical performances were investigated by cyclic voltammetry and galvanostatic charge–discharge measurements.

## 2. Results and Discussions

**Figure 3:**
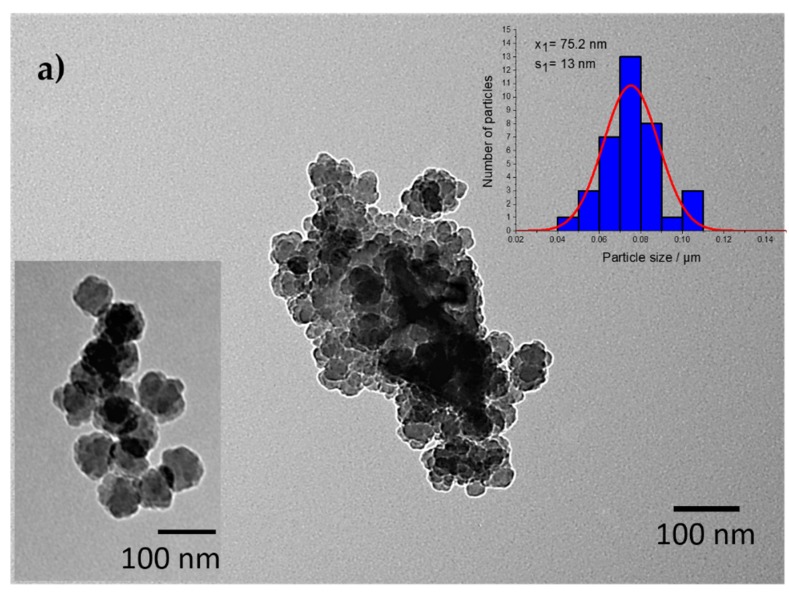

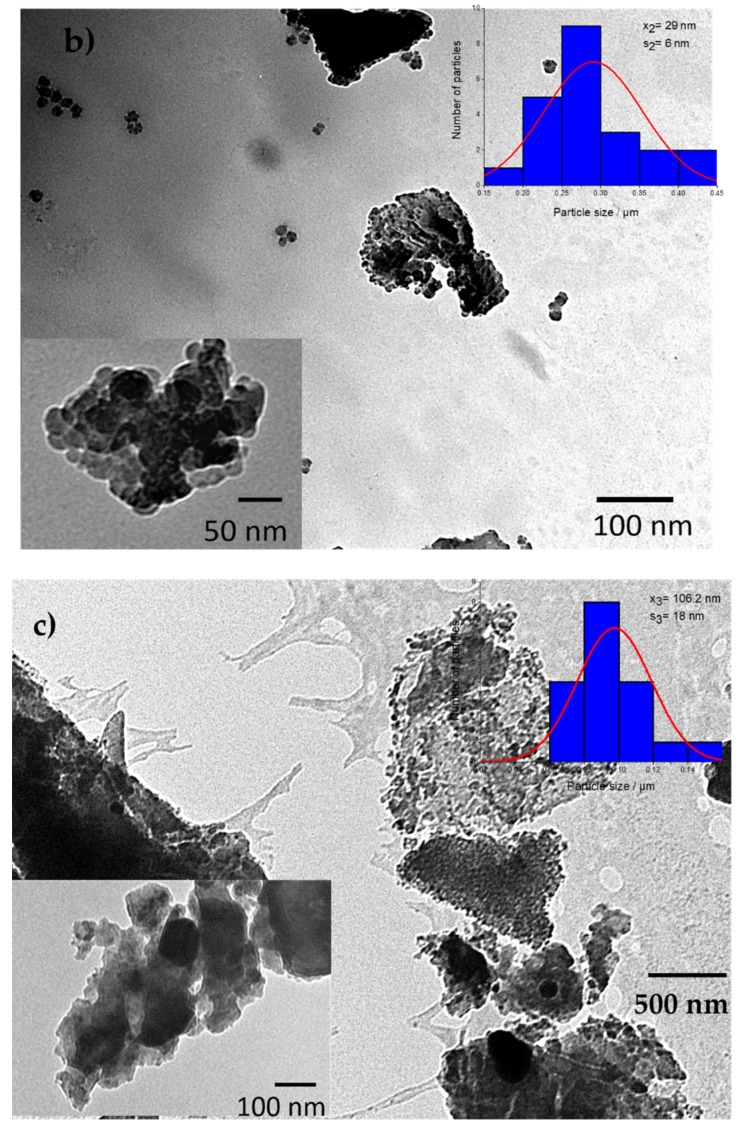
TEM images of samples (**a**) S1, (**b**) S2, and (**c**) S3 with calculated particle size distributions.

Figure 3 shows TEM images of powder samples S1 (a), S2 (b), and S3 (c). The TEM image of sample S1 shows agglomerates composed of a dozen smaller particles. The measured particle size distribution of sample S1 gave the mean particle size of ~75.2 nm (+/− 13 nm), while the size of the agglomerate proved to be much larger (approximately 250 nm). The ball-milled sample (S2) possesses of small discrete particles around 29 nm (+/− 6 nm) in size and bigger particle agglomerates approximately 200 nm in size. Sample S3 consisted of irregular particles (c) with a lot of empty spaces between particles, which indicated the porous nature of sample S3. The particle size of ~106.2 nm (+/− 18 nm) and particle agglomerate size of approximately 500 nm were measured (c).

**Figure 4:**
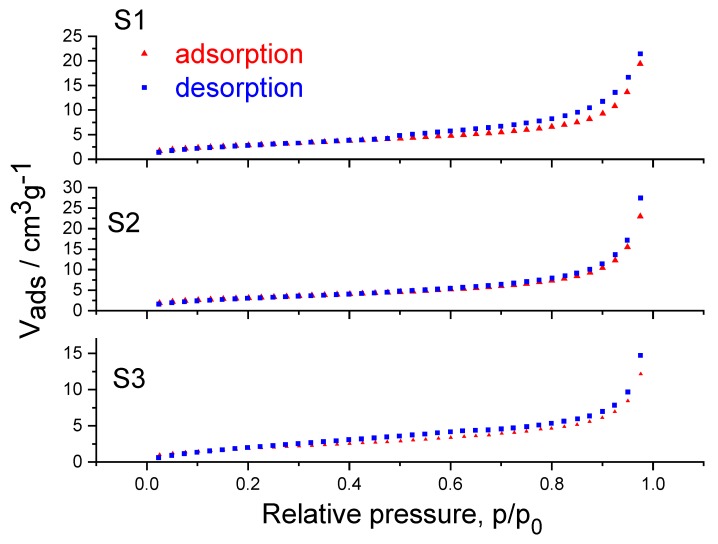
Gas (N_2_) adsorption (red line, triangles) and desorption (blue line, squares) isotherms.

The total volume and specific surface areas of samples were measured using nitrogen adsorption. For these analyses, the samples were degassed at T = 250 °C and the sample chamber was filled with controlled increments of nitrogen starting at relative pressure of p/p_0_ = 0.025 at T = 77K, where p_0_ is the saturation vapor pressure of liquid N_2_ at 77K. Figure 6 shows the N_2_ adsorption-desorption isotherms of the samples S1, S2, and S3. Samples S1 and S3 can be characterized by the H4 type hysteresis loops of physisorption isotherms. These types of loops are generally found within materials possessing narrow slit pores, often in the micropore region. For sample S1, the hysteresis loop closes at around 0.47 p/p_0_ which is indicative of the so-called tensile strength effect. In contrast to the hysteresis loop of sample S1, the hysteresis loop of sample S3 is observable in the low-pressure region, which can be a consequence of irreversible adsorption of nitrogen in pores of a similar width as the adsorptive molecule or the chemisorption of the adsorptive molecules. Due to the presence of the low-pressure hysteresis in sample S3, an accurate analysis of pore size and pore volume is not possible. In contrast to these two samples, S2 exhibits a type II physisorption isotherm, which is characteristic of nonporous or macroporous adsorbents. The shape of such an isotherm is a consequence of unrestricted monolayer-multilayer adsorption up to high values of p/p_0_ [44,45], which is in line with particle agglomeration visible on the TEM images. The difference in the physisorption isotherms (Figure 4) is a result of mechanochemical treatment of sample S2 which can influence the structure of the samples regarding both micropores and mesopores [46]. From these data, the total surface area of the sample can be derived by the method of Brunauer, Emmet and Teller (BET) [47]. For sample S1 the surface area is 10.6 m^2^/g, while for S2 it is 16.7 m^2^/g. The surface increase is expected because the particle size is reduced. Sample S3 has a surface area of 7.2 m^2^/g which is also expected because the average pore size of S3 (3.44 nm) is much bigger than for the S2 (1.26 nm) or S1 (0.56 nm). The total pore volume increased in sample S3. The average particle size and surface area of samples S1, S2, and S3 are given in Table 1.

**Figure 5:**
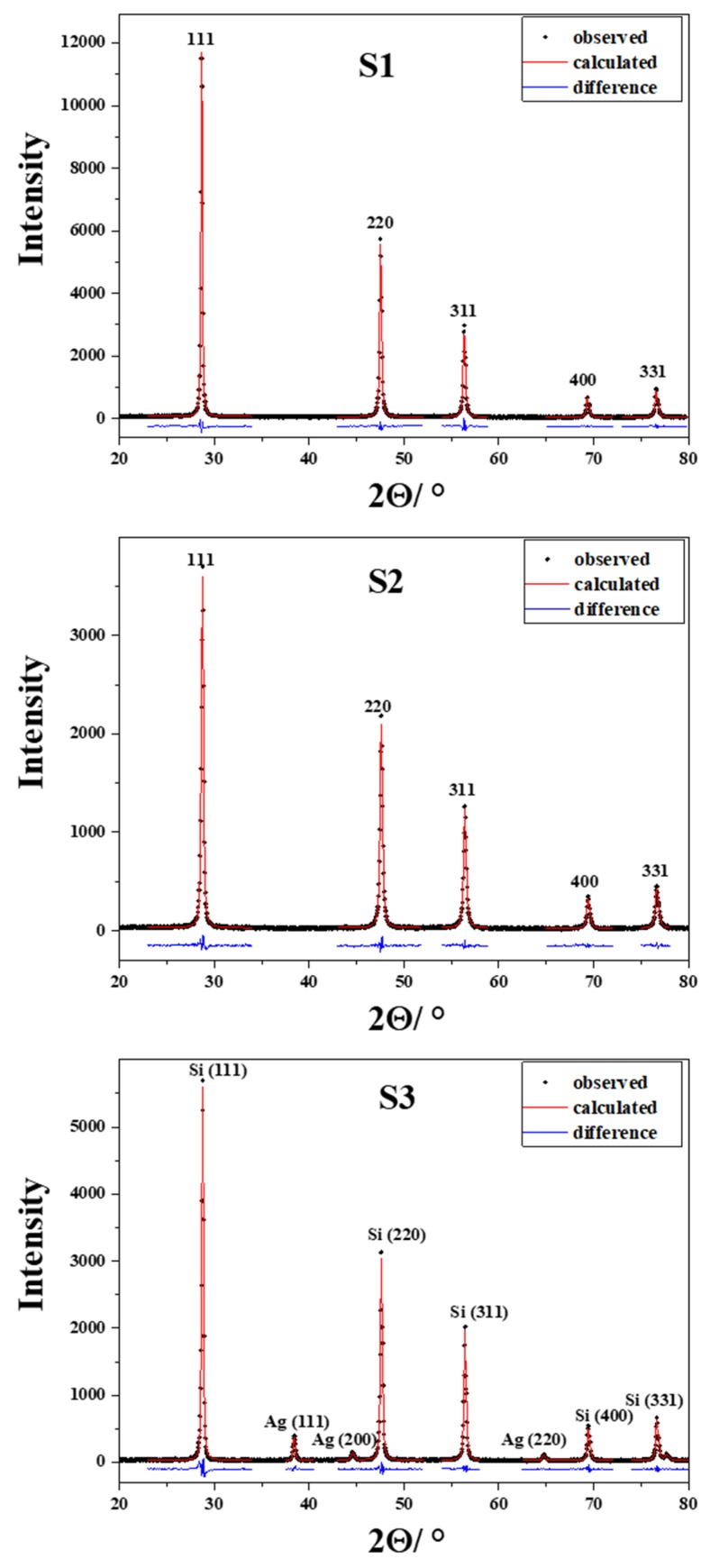
X-ray powder diffraction (XRD) patterns of samples S1, S2, and S3.

**Figure 6:**
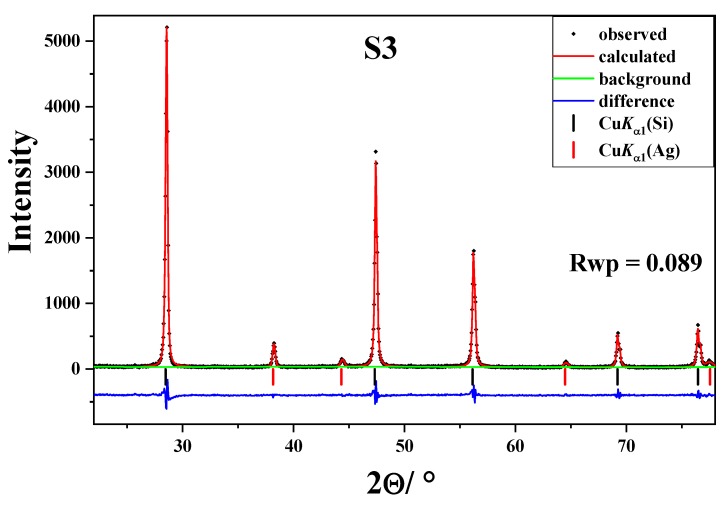
Rietveld refinement of powder diffraction patterns for sample S3.

Figure 5 shows the X-ray powder diffraction patterns of samples S1, S2, and S3. The X-ray powder diffraction patterns of these samples contain only diffraction lines typical of silicon (Si, space group *Fm*$\bar{3}$*m*, a = 5.43088 Å; ICDD PDF No. 27-1402). In the case of sample S1, diffraction lines are very strong and narrow, which indicate a well crystalline sample. Milling of sample S2 caused a broadening of the diffraction lines and the increase of background due to the decrease of crystallite size and the increase of lattice defects. The X-ray powder diffraction pattern of S3 contains, beside strong and narrow diffraction lines typical of silicon, diffraction lines of lower intensity typical of silver (Ag, space group *Fm*$\bar{3}$*m*, a = 4.0862 Å; ICDD PDF No. 4-783). During the processing of various materials in the mill, in addition to grinding particles and forming defects, the metastable phases might be formed [48]. In particular, the small quantities of high-pressure phases were formed upon silicon milling [49]. In order to determine portion of crystal phases (silicon and silver) in sample S3, we performed Rietveld refinement of a powder diffraction pattern (Figure 6). The obtained results indicate that this sample is predominantly silicon (~99.5%). The amount of the second crystalline phase, silver, was estimated at ~0.5%. The weighted residual error index (R_wp_) of the refined pattern was less than 9%.

The physical broadening (β) of the diffraction lines was used to estimate the volume-averaged domain size (*D*_v_) and the root-mean-square strain (*ε*_RMS_) according to the so-called ”double-Voigt” method [50] equivalent to the Warren-Averbach approach [51]. This method, in which Voigt functions were used to describe the contribution of both the crystallite size and the lattice micro-strain to the broadening of the diffraction lines, was performed using the computer program BREADTH [52]. Physically broadened diffraction line profiles (β) were obtained by convolution-fitting approach (program SHADOW [53]) in which the instrumental profile (a split Person VII function fitted to the very narrow diffraction lines of zincite [54]) is convoluted with a refineable Voigt function to fit the observed diffraction lines profile. The results of convolution-fitting of the diffraction lines in products S1, S2, and S3 are shown in Figure 5. The results of diffraction line broadening analysis are summarized in Table 2.

**Figure 7:**
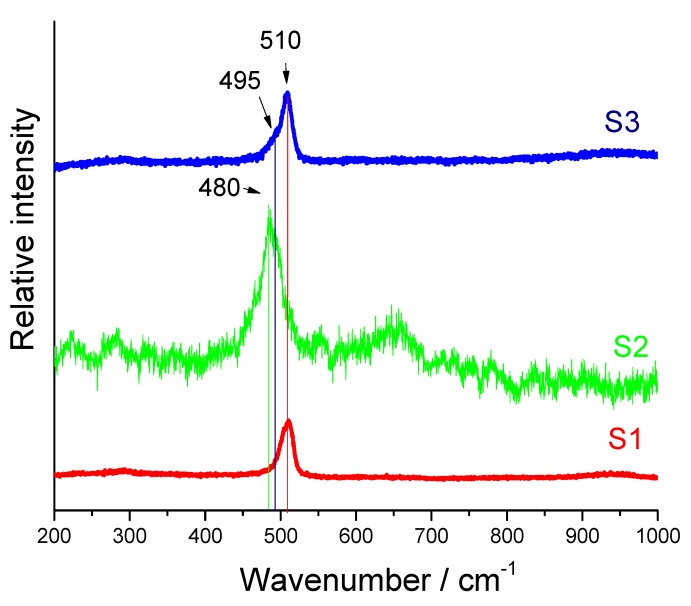
Raman spectra of samples S1, S2, and S3.

Figure 7 shows Raman spectra of samples S1, S2, and S3. The Raman peak at 510 cm^−1^ (~40 cm^−1^ width) found in sample S1 is characteristic for the crystalline silicon (c-Si) powder. The mechanochemical treatment of the sample produced defects and strain and as a consequence the c-Si peak at 510 cm^−1^ was further shifted toward lower wavenumbers by 30 cm^−1^, and it was further broadened by an additional ~10 cm^−1^. These changes indicate that the lattice has been further defected and strained, resulting in a change of the Si–Si bond length [55,56]. Sample S2 also shows photoluminescence (PL) as a result of new formatted Si-O bonds in the sample as shown on the FT-IR spectrum. The PL of porous silicon is attributed to the recombination centers related to Si-O bonds. The phonon confinement can be explained by the formation of the nanocrystalline silicon domains created by mechanochemical treatment. The Raman spectrum of sample S3 contains a peak at 510 and shoulder at 495 cm^−1^. The origin of the first one is due to phonon confinement in silicon nanoparticles and the second one is due to amorphous silicon layers or surface phonons of silicon nanoparticles [57,58]. The increased number of silicon nano-domains could be caused by MACE treatment. There is no observable PL effect in the sample S3 because the MACE process could remove the oxide layers by chemical etching.

**Figure 8:**
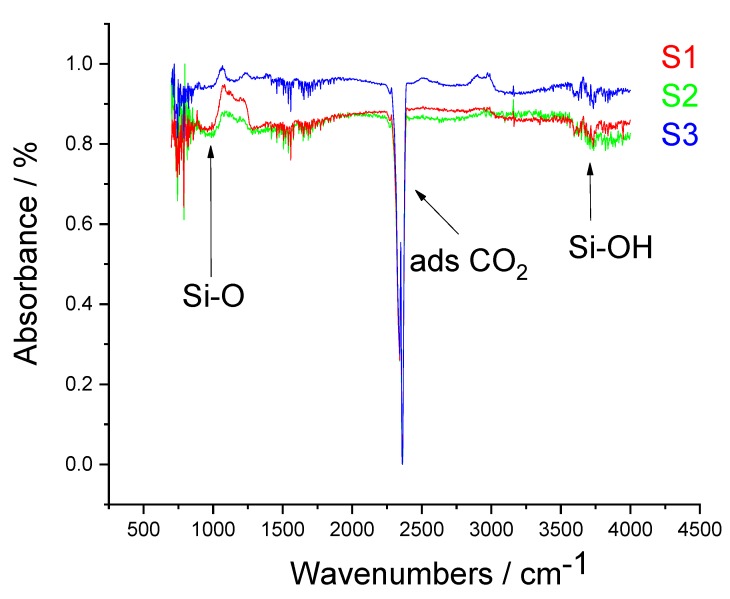
FT-IR spectra of samples S1, S2, and S3.

Figure 8 shows FT-IR spectra of samples. All three samples show a broad band ranging from 3700 to 3200 cm^−1^ associated with the stretching mode of hydrogen in Si-OH groups. A strong band at 2280–2080 cm^−1^ is in the range of silicon hydrogen species (SiH_x_, x = 1–3) [59], but due to the characteristic shape of the band, we assigned this band to the adsorbed CO_2_. The characteristic asymmetric stretching mode for Si-O corresponds to the band in the range of 1100 to 1400 cm^−1^ for samples S1 and S2, which is in line with the PL effect present in the Raman spectrum of S2 [59].

**Figure 9:**
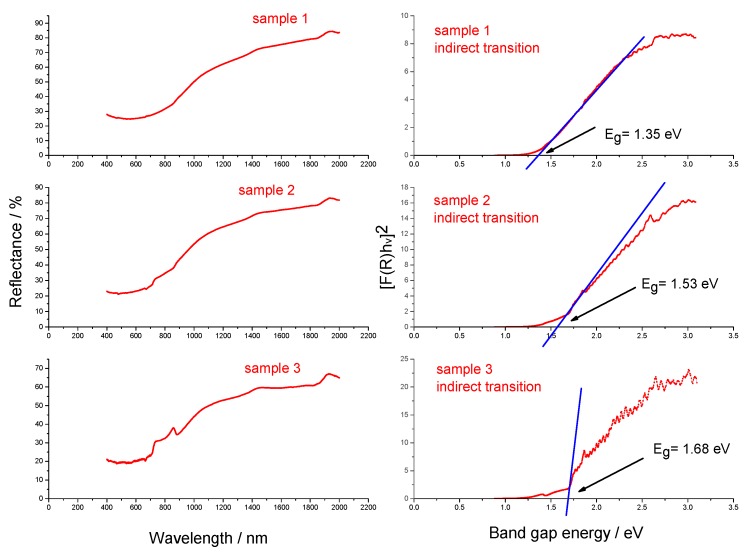
UV-Vis diffuse reflectance spectra of samples S1, S2, and S3 (left panel) and the corresponding Kubelka–Munk plots (right panel).

Figure 9 shows UV-Vis diffuse reflectance spectra (a) of samples S1, S2, and S3 and the corresponding Kubelka-Munk plots (b). The band gap energy of samples S1, S2, and S3 was calculated based on the Kubelka-Munk function. The following relation is used:

(*hνα*)^1/n^ = *A* (*hν* − *E*_*g*_) (1)

where *h* is Planck’s constant, *ν* is frequency, *α* is absorption coefficient, *E*_*g*_ is band gap and A is proportionality constant. The value of the exponent n represents the type of sample transition (n = 2 for indirect allowed transition, n = 0.5 for direct allowed transition). The calculations were performed for indirect band gap energy determination; therefore, the value of n was set to 2. The collected diffuse reflectance spectra were converted to the Kubelka–Munk function. The vertical axis was converted to a quantity (*R*∞)*hν* where *F*(*R*∞) is proportional to the absorption coefficient and is calculated by the following equation:

*F*(*R*∞) = (1 − *R*)^2^/2*R* (2)

where *R* is reflectance at a given wavelength. Using the calculated values, *F*(*R*∞) *hν*^2^ was plotted against *hν*. A line tangent to the linear part of the curve was extrapolated to zero reflectance. The extrapolated value was taken as the band gap energy of the material. The calculated band gaps (*E_g_*) of samples S1, S2, and S3 were estimated to 1.35, 1.53, and 1.68 eV, respectively which is in agreement with Raman scattering measurements that show silicon nanosized domains for all samples analyzed. Band gap increases with the decrease in size due to electron confinement at the nano scale.

**Figure 10:**
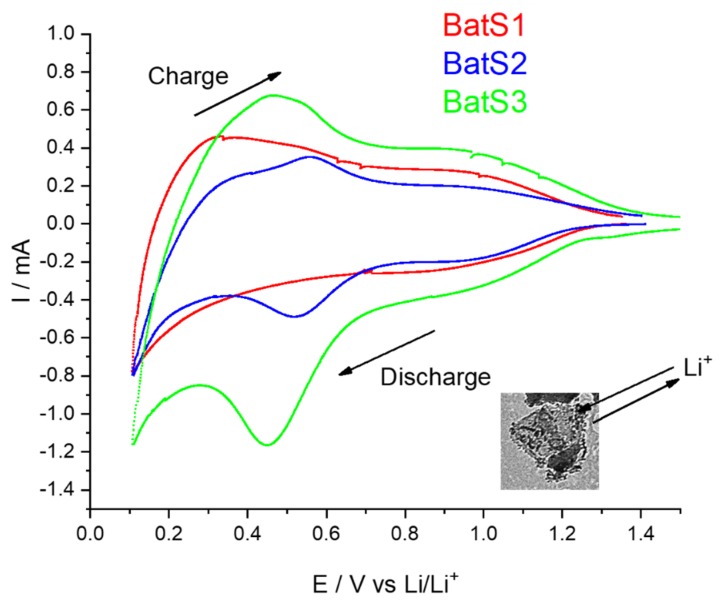
Cyclic voltammetry of samples BatS1-, BatS2-, and BatS3-based anode material in LP40 (1M LiPF_6_ in 1:1 ethylene carbonate (EC): dimethyl carbonate (DMC)) with Li foil as a counter and reference electrode at room temperature and scan rate 1 mV/s.

**Figure 11:**
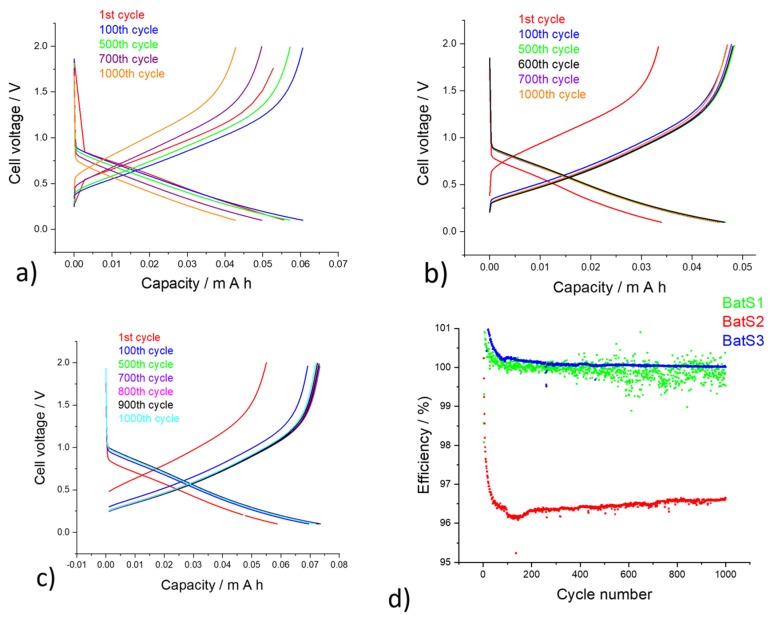
Galvanostatic charge-discharge curves of (**a**) BatS1, (**b**) BatS2, (**c**) BatS3, and (**d**) efficiency of all three battery cells.

Electrochemical measurements were performed using both the cyclic voltammetry (CV) (Figure 10) and galvanostatic cycling (Figure 11). The intercalation and extraction mechanism are followed by CV, which indicates, for sample BatS1, the absence of Li^+^ ion diffusion through anode material. On the contrary, initial sweep cycle of samples BatS2 and BatS3, shows cathodic peak (Li alloying), current increases sharply below 0.5 V vs. Li/Li^+^, which is related to intercalation of Li^+^ ions resulting in the transformation of crystalline Si to a disordered phase [58,60]. In the reverse (discharge) process, a broad anodic peak is due to the extraction of Li^+^ ion from Li_x_Si regenerating to Si. Two anode peaks at BatS2 and BatS3, characteristic for extraction of lithium ion from silicon anode, indicate that Li^+^ ion is extracted from different crystalline phases. Sample BatS3 shows much higher charge values compared to samples BatS1 and BatS2. Moreover, the current values for sample BatS3 were higher for several orders of magnitude in comparison to samples BatS1 and BatS2.

**Figure 12:**
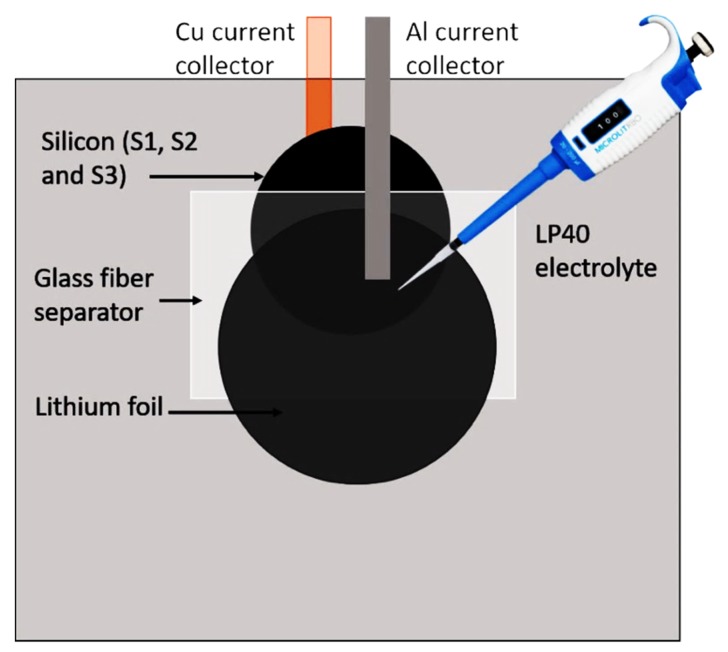
“Coffee bag“ battery assembly.

Galvanostatic charge/discharge measurements show intercalation and extraction cycles which demonstrates good stability, which can undergo over 100 cycles for sample BatS1, 500 cycles for sample BatS2 and 700 cycles for sample BatS3 (Figure 12). The efficiency of the BatS1 is quite diffuse, for sample BatS2 is slightly smaller and more stable over a longer cycle, whereas for sample BatS3 it has higher efficiency and much longer stability over many cycles. This allows us to conclude that sample S3 may indeed provide a good quality anode material.

According to Ikonen et al., porous silicon was prepared by electrochemical etching of silicon wafers and ball milling. The average pore size of pSi was 5–16 nm and the obtained electrode shows a capacity of 1200 mAh/g [28]. According to Gauthier et al., an Si-based anode with improved performance was achieved using high-energy ball milling as a cheap and easy process to produce Si powder. Milled powders were nanostructured with micrometric agglomerates (approximately 10 µm in size), made of submicrometric particles with crystallite size of 10 nm. They showed that, compared to non-milled 1–5 µm powders, the improved performance is linked to a strong lowering of particle disconnection at each charge, while the irreversibility due to solid electrolyte interphase (SEI) formation remains unchanged. Electrode achieves 600 cycles at more than 1170 mAh/g with coulombic efficiency above 99% [61]. In the study by Zhang et al., one-dimensional porous silicon nanowires were prepared through the MACE process by using metallurgical silicon as raw material. Nanowires were coated with crossed carbon skeleton via in situ polymerization and carbonization process. The resultant composite delivered a high capacity of 1253 mAh/g with good cycling stability [43]. In this work, metal-assisted chemical etching of starting Si material resulted in the average pore size of 3.44 nm, which is large enough for the solvated lithium ions to pass through pores easily.

## 3. Materials and Methods

### 3.1. Sample Preparation

#### 3.1.1. Sample S1

Commercial micrometric silicon powder, denoted as sample S1, was used as the starting material.

#### 3.1.2. Sample S2

Sample S2 was prepared by milling sample S1. Milling was performed in air using a Fritsch planetary ball mill “Pulverisette 6” with stainless steel (18% Cr + 8%Ni) milling assembly. The rotation speed was 400 rpm, and the powder-to-ball weight ratio was 1:10. Milling time was 30 min.

#### 3.1.3. Sample S3

Sample S3 was porous silicon nanowires (pSi-NWs) synthesized through metal-assisted chemical etching (MACE) following the procedure of Zhang et al. [43] using sample S1 as feedstock. Si powder was degreased in a Diener Electronic ZEPTO plasma cleaner under O_2_ for 5 min. Then, 1 g of sample S1 were added to a 100 mL solution of 75 mL H_2_O, 25 mL HF (5M) and 0.344 g AgNO_3_. The Ag plating proceeded at room temperature for 5 min. After the first step (HF/AgNO_3_) of etching, we observe the formation of small silver particles on the surface of silicon particles. These particles grew in size and formed silver dendrites. During the first minutes of etching in the second solution (HF/H_2_O_2_), the silver particles sink into the substrate, the silver dendrites grow on and the nanowires are formed as the remaining of the unetched silicon [58]. Silver-deposited Si particles were etched in 5M HF and 0.3 M H_2_O_2_ at room temperature. The metal-assisted chemical etching was performed for 8 min in an ultrasonic bath and then stopped by adding a large amount of deionized water to the etching solution. The etched Si powder was filtered through a micropore filter paper and rinsed several times with deionized water. The Ag particles loaded on the etched Si powder were easily removed with standard Ag etchant (NH_4_OH:H_2_O_2_ = 3:1 (*v*/*v*)). The product, denoted as sample S3, was rinsed with deionized water and dried.

#### 3.1.4. Electrode Preparation

**Figure 13:**
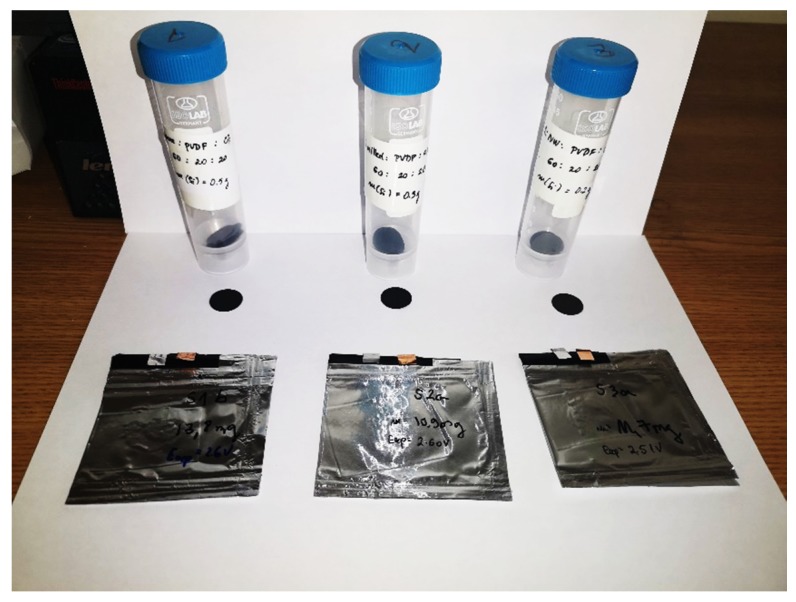
Prepared electrodes with associated “coffee bag” batteries.

For the preparation of Si-based anodes, we used powder silicon samples (S1, S2, and S3) mixed with polyvinylidene fluoride (PVDF) and conductive carbon black (CB) in N-Methyl-2-pyrrolidone (NMP). The ratio applied was 60 wt-% of samples S1, S2, and S3, 20 wt-% of PVDF, and 20 wt-% of CB. The prepared slurries were then spread onto aluminum foil current collector (20 µm thick) using a Doctorblade coating machine for wet thickness of 200 µm. The prepared electrode sheets were dried for 12 h at 60 °C in vacuum. Thereafter, circular electrodes with 16 mm diameter (A = 2 cm^2^ ) were cut with an electrode punching tool and placed in a vacuum oven over night at 50 °C. The typical loading of composite electrode material was ≈12 mg. Figure 13 shows prepared electrodes with associated “coffee bag” batteries.

#### 3.1.5. Battery Assembly

The “coffee bag“cells were assembled in an argon glove box. The electrolyte was LP40 (1M LiPF_6_ in 1:1 ethylene carbonate (EC): dimethyl carbonate (DMC)). The obtained battery cells were denoted as BatS1, BatS2, and BatS3.

### 3.2. Instrumental Analysis

The morphology of the obtained materials was studied by transmission electron microscope (Jeol JEM 1010) (Tokyo, Japan). XRD measurements were taken in a step-scan mode using an ItalStructures diffractometer APD2000 (Riva del Garda, Italy) with monochromatized CuKα radiation (graphite monochromator). XRD patterns were scanned in 0.05° steps (2*Ѳ*), in the 2*Ѳ* range from 10° to 80°, with a fixed counting time (15 s). In cases where more than one crystalline phase coexists in the sample, we provided a quantitative analysis using Rietveld refinements of powder diffraction patterns [62] in order to determine the share of each crystal phase in the sample. For Rietveld refinements, we used the computer program MAUD [62].

Raman spectra were recorded using a Horiba Jobin Yvon T64000 micro Raman system (laser wavelength of 532 nm) (Lille, France). The experiments were done at room temperature and a working power of 0.7 W. FT-IR spectroscopy was applied to identify the characteristic peaks and it was recorded using a Nexus 470 FT-IR Nicolet with an AVATAR OMNI-Sampler (Madison, WI, USA). UV-Vis spectra were collected with a Shimadzu UV/VIS/NIR spectrometer (Tokyo, Japan), model UV-3600. The used wavelength range was from 2000 to 400 nm.

The surface structure parameters of the samples were determined using N_2_ adsorption–desorption measurements. The adsorption–desorption isotherms were measured by an Autosorb iQ-AG-C Quantachrome instrument (Boyton Beach, FL, USA) at 77 K. The Brunauer–Emmet–Teller (BET) method was used to determine the specific surface area.

The results of capacitive properties and stability were determined using cyclic voltammetry (CV) with a Solartron SI 1287 electrochemical interface and galvanostatic charge-discharge by BioLogic SP-200. Cyclic voltammetry of samples BatS1, BatS2, and BatS3 was performed in an LP40 (1M LiPF_6_ in 1:1 ethylene carbonate (EC): dimethyl carbonate (DMC)) as electrolyte, glass fiber separator and Li foil as counter and reference electrode at room temperature and scan rate 1 mV/s. To assess the charge/discharge capability the electrodes were galvanostatically cycled between 2.0 and 0.1 V vs. Li/Li ^+^.

## 4. Conclusions

The microstructural and physico-chemical properties of silicon microparticles were studied as a promising and non-expensive lithium ion battery anode material. The commercial silicon powder was used as a starting material. The ball-milling silicon powder resulted in the negligible enhancement of average pore and silicon domain size. The metal-assisted chemical etching (MACE) of starting Si material resulted in the significant increase of average pore size (from 0.56 to 3.44 nm), which is large enough for the solvated lithium ions to pass easily through the pores. The large pore size makes the MACE sample very promising for use as a lithium ion battery anode material, which is confirmed by the cyclic voltammetry and galvanostatic cycling measurements. The MACE treated sample BatS3 possessed the highest charge value and good efficiency over 700 cycles.

## Figures and Tables

**Table 1:** The average particle size (TEM) and surface area of samples (BET method).

Sample	Average Particle Size/nm	Surface Area/m^2^g^−1^
S1	~75.2 nm (+/− 13)	10.6
S2	~29 nm (+/− 6)	16.7
S3	~106.2 nm (+/− 18)	7.2

**Table 2:** Values of volume-averaged domain size (*D*_v_) and the root-mean-square strain (RMSS) of Si and Ag crystal phases in the samples S1, S2, and S3 as determined by the double-Voigt method [50].

Sample	Phase	Double-Voigt Method	
		*D*_v/_nm	RMSS × 10^3^
S1	Si	49(2)	0.98(6)
S2	Si	32(1)	1.20(8)
S3	Si	35(1)	0.97(5)
	Ag	33(2)	1.36(18)
